# Transverse effect of Haas and Hyrax appliances on the upper dental arch in patients with unilateral complete cleft lip and palate: A comparative study

**DOI:** 10.1590/2176-9451.19.2.039-045.oar

**Published:** 2014

**Authors:** Anna Júlia de Oliveira Façanha, Tulio Silva Lara, Daniela Gamba Garib, Omar Gabriel da Silva Filho

**Affiliations:** 1 Specialist in Orthodontics, Hospital for Rehabilitation of Craniofacial Anomalies - São Paulo University (HRAC-USP); 2 Professor of Interceptive Orthodontics (HRAC-USP); 3 Full professor, School of Dentistry - University of São Paulo/Bauru. Professor of Orthodontics (HRAC-USP); 4 State University of São Paulo, MSc in Orthodontics, State University of São Paulo (UNESP)

**Keywords:** Palatine expansion technique, Cleft palate, Dental arch

## Abstract

**Objective:**

The aim of the present study was to evaluate the transverse effect of rapid maxillary expansion in patients with unilateral complete cleft lip and palate while comparing the Haas and Hyrax appliances.

**Methods:**

The sample consisted of 48 patients divided into two groups: Group I - 25 patients treated with modified Haas appliance (mean age: 10 years 8 months); and Group II - 23 patients treated with Hyrax appliance (mean age: 10 years 6 months). Casts were taken during pre-expansion and after removal of the appliance at the end of the retention period. The models were scanned with the aid of the 3 Shape R700 3D scanner. Initial and final transverse distances were measured at cusp tips and cervical-palatal points of maxillary teeth by using the Ortho Analyzer^TM^ 3D software.

**Results:**

The mean expansion obtained between cusp tips and cervical-palatal points for inter-canine width was 4.80 mm and 4.35 mm with the Haas appliance and 5.91 mm and 5.91 mm with the Hyrax appliance. As for first premolars or first deciduous molars, the values obtained were 6.46 mm and 5.90 mm in the Haas group and 7.11 mm and 6.65 mm in the Hyrax group. With regard to first molars, values were 6.11 mm and 5.24 mm in the Haas group and 7.55 mm and 6.31 mm in the Hyrax group.

**Conclusion:**

Rapid maxillary expansion significantly increased the transverse dimensions of the upper dental arch in patients with cleft palate, with no significant differences between the Hass and Hyrax expanders.

## Abstract (pt)

**Objetivo:**

avaliar o efeito transversal na arcada dentária superior do procedimento de expansão rápida da maxila em pacientes com fissura transforame incisivo Aunilateral, comparando os expansores tipo Haas modificado e de Hyrax.

**Métodos:**

a amostra constou de 48 pacientes divididos em dois grupos: grupo I, 25 pacientes que utilizaram o aparelho expansor tipo Haas modificado, com média de idade de 10 anos e 8 meses; e grupo II, 23 pacientes que utilizaram o Hyrax, com média de idade de 10 anos e 6 meses. Modelos de gesso foram realizados na fase pré-expansão e após 6 meses de contenção, após a remoção do aparelho. Os modelos foram digitalizados com auxílio do *scanner* 3Shape R700 3D e as distâncias transversais iniciais e finais foram medidas entre as pontas de cúspides e pontos cervicopalatinos de dentes superiores pelo método digital no *software* OrthoAnalyserT 3D.

**Resultados:**

a média de expansão obtida entre as pontas de cúspides e entre os pontos cervicopalatinos, respectivamente, para a distância intercaninos, foi de 4,80mm e 4,35mm para o Haas e de 5,91mm e 5,91mm para o Hyrax; 6,46mm e 5,90mm para os primeiros molares decíduos ou primeiros pré-molares no grupo Haas, 7,11mm e 6,65mm no grupo Hyrax; e 6,11mm e 5,24mm para os primeiros molares no grupo Haas e 7,55mm e 6,31mm no grupo Hyrax.

**Conclusão:**

o procedimento de expansão rápida da maxila produziu aumentos significativos das dimensões transversais da arcada dentária superior em pacientes com fissura, sem diferenças significativas entre os expansores Haas modificado e Hyrax.

## INTRODUCTION

Unilateral complete cleft lip and palate simultaneously involves the primary and secondary palate and accounts for 30% of all clefts. This condition requires more extensive treatment, as the cleft divides the maxilla and the alveolar arch into two completely distinct segments.^1^ Treatment initially involves primary functional and esthetic surgeries for the closing of the lip and palate, which have a long-term impact on mid face growth.^2,3^ The patient is then followed up throughout the growth period until entering the orthodontic phase (end of the deciduous dentition phase).^4^

Primary surgeries of the lip and palate usually potentiate reductions in the transverse and sagittal dimensions of the upper arch as a consequence of the restricted growth of the mid face and the approximation of the initially separated maxillary segments.^3^ These sagittal and transverse deficiencies of the upper alveolar arch is expressed already at the mixed dentition phase and tend to become aggravated in adolescence.^5^ Thus, the task of orthodontists is to counteract the harmful effects of the altered facial growth also characterized by posterior and anterior cross bite - often found in patients with cleft lip and palate.^6^

Besides other occlusion issues,^7^ posterior cross bite is the most common malocclusion in these patients, involving a single tooth or the entire dental arch, showing a tendency toward exacerbation from the mixed to the permanent dentition. Interceptive orthodontic interventions should be performed during the mixed dentition phase for correction of the compromised transverse dimension.^8^ Moreover, the expansion of the upper arch also plays an important role in preparing the arch and cleft region for the secondary alveolar bone graft, which is performed at the end of the mixed dentition prior to the eruption of the permanent canine adjacent to the cleft region.^4^

Rapid maxillary expansion performed by means of a Haas or Hyrax appliance is the most common method employed at the Hospital for Rehabilitation of Craniofacial Anomalies (USP, Brazil) to increase the width of the maxilla. The main difference between the two appliances is the type of anchorage: tooth-supported with the Hyrax appliance and tooth-mucosa-supported with the Haas appliance.

The active expansion phase in patients with unilateral complete cleft lip and palate promotes distancing of the maxillary segments and widening of the cleft.^8^ Expansion in these patients is not followed by bone formation at the median palatine suture, as it occurs in patients without cleft palate,^9^ because the distancing of the maxillary halves occurs in the region of the cleft. Thus, retention should remain after the appliance is removed and until bone graft is performed. Expansion in such patients involves similar restrictions to those found in patients without cleft palate, as the other maxillary sutures offer considerable resistance to widening, requiring orthopedic appliances.

Motivated by the interest in evaluating the results of the expansion philosophy of the team at the Hospital for Rehabilitation of Craniofacial Anomalies/USP, the aim of the present prospective study was to assess alterations in the transverse dimension of the upper dental arch in patients with unilateral complete cleft lip and palate who have undergone rapid maxillary expansion, comparing the results achieved with the Haas and Hyrax appliances with the aid of digital models.

## MATERIAL AND METHODS

This study was approved by the Hospital for Rehabilitation of Craniofacial Anomalies/USP Institutional Review Board (protocol 255/2010-SVAPEPE-CEP).

The sample consisted of 48 patients enrolled in the orthodontic sector of the hospital. All patients had unilateral complete cleft lip and palate, had undergone primary surgeries at an early age and were in the mixed dentition phase, exhibiting maxillary atresia with an indication for rapid maxillary expansion.

**Figure 1:**
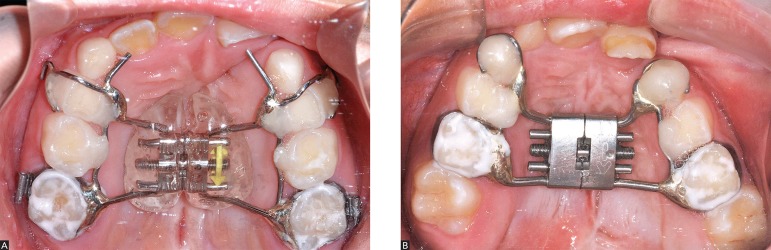
Occlusal photographs of two patients from the sample illustrating both expanders: Modified Haas (A) and Hyrax (B)

The patients were randomly divided into two groups. Group I comprised 25 patients treated with the modified Haas appliance (Fig 1A), with bands on the permanent molars and orthodontic clip bonded to the deciduous molars (16 males and 9 females; mean age: 10 years 8 months; age range: 8 years to 19 years and 2 months). Group II comprised 23 patients treated with the Hyrax appliance (Fig 1B) (13 males and 10 females; mean age: 10 years 6 months; age range: 8 years and 2 months to 18 years and 1 month). All patients underwent the same activation protocol. The expander was activated for seven days with 2/4 turns in the morning and 2/4 turns in the evening. Whenever necessary, further activation was performed until overcorrection was reached (palatine cusps of the upper molars occluding the vestibular cusps of the lower molars). Hooks for a facial mask were welded to the inverse traction of the maxilla for patients that also had sagittal discrepancy with good prognosis for orthopedic treatment.

After achieving overcorrection, the screw was stabilized with acrylic resin. The expander was maintained passive with a retention protocol for six months, after which the expander was removed and a fixed retainer was installed.

**Figure 2:**
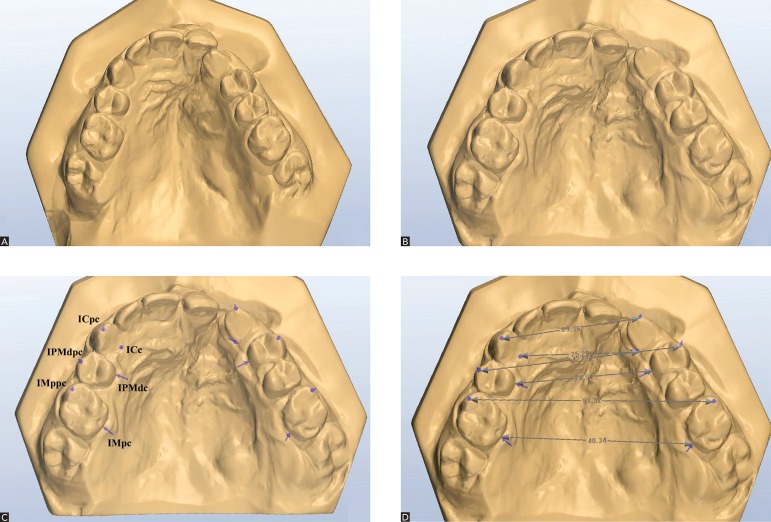
Initial and final digital models showing the results of rapid maxillary expansion (A and B); reference points (C) and measurements made (D) on postexpansion models

Dental cast of the maxillary arch were obtained at two different times: immediately before banding for the expander (T_1_), and after the six-month period of retention immediately following the removal of the expander (T_2_). The impressions were made with alginate and filled with Paris plaster. The dental casts were then scanned by a 3Shape R700 3D scanner (3Shape A/S, Copenhagen, Denmark), which reproduces a three-dimensional digital image based on laser beams scans projected over the plaster models in different directions. The Ortho Analyzer ^TM^ 3D program was used to obtain the three-dimensional image of the scanned models, allowing frontal, lateral, posterior and occlusal visualization. The reference points were marked on the model in the occlusal view for the calculation of the distances between teeth. Figure 2 illustrates the measurements made on the digital models of the upper dental arches.

Analyses were performed by a single examiner. Sixty percent of the sample was analyzed a second time after a seven-day interval in order to determine the measurements reliability using the intraclass correlation coefficient (ICC) for which the following scores were used: 0.80 to 1.00 = excellent agreement; 0.60 to 0.80 = substantial; 0.40 to 0.60 = moderate; 0.20 to 0.40 = fair; 0 to 0.20 = discreet; and -1 to 0 = poor.

Mean, standard deviation (SD), maximum and minimum values were calculated. The paired Student's t-test was employed to determine statistically significant differences between the initial and final measurements of each group. The independent t-test was used for inter-group comparison. The level of significance was set at 5% (p < 0.05).

## RESULTS

Tables 1 to 4 display the results of the measurement reliability and transverse distances measured on the models.

**Table 1:** Error of the method (Intraclass Correlation Coefficient).

Measure	ICC (Haas)		ICC (Hyrax)	
	Initial	Final	Initial	Final
ICpc	0.99	0.82	1.00	0.99
ICc	0.97	0.99	0.99	1.00
IPMdpc	0.91	1.00	0.99	1.00
IPMdc	0.99	0.99	1.00	1.00
IMppc	0.99	0.98	0.96	0.99
IMpc	1.00	0.99	1.00	0.99

**Table 2:** Transverse distances measured on initial and final digital models and mean amount of expansion (Dif.) in millimeters for patients using the modified Haas expander.

Measure	Initial model	Final model	Dif.	p
	Mean ± S.D.	Mean ± S.D.		
ICpc	26.81 ± 3.13	31.60 ± 3.35	4.79	< 0.01*
ICc	21.15 ± 3.39	25.50 ± 3.76	4.35	< 0.01*
IPMdpc	35.39 ± 3.67	41.85 ± 4.22	6.46	< 0.01*
IPMdc	24.25 ± 3.03	30.15 ± 3.36	5.9	< 0.01*
IMppc	49.20 ± 3.91	55.31 ± 3.28	6.11	< 0.01*
IMpc	35.29 ± 2.84	40.54 ± 3.24	5.24	< 0.01*

* statistically significant difference (p < 0.05)

**Table 3:** Transverse distances measured on initial and final digital models and mean amount of expansion (Dif.) in millimeters for patients using the Hyrax expander.

Measure	Initial model	Final model	Dif.	p
	Mean ± S.D.	Mean ± S.D.		
ICpc	24.63 ± 3.98	30.54 ± 2.95	5.91	< 0.01*
ICc	20.37 ± 2.96	26.28 ± 3.91	5.91	< 0.01*
IPMdc	33.10 ± 3.71	40.21 ± 3.96	7.12	< 0.01*
IPMdc	22.72 ± 3.59	29.37 ± 3.50	6.65	< 0.01*
IMppc	44.68 ± 4.26	52.23 ± 3.73	7.55	< 0.01*
IMpc	31.32 ± 3.90	37.63 ± 3.79	6.31	< 0.01*

* statistically significant difference (p < 0.05)

**Table 4:** Comparison of modified Haas and Hyrax appliances groups regarding mean increase in transverse dimensions of the maxilla following rapid maxillary expansion.

Measure	Group Haas (n)	Group Hyrax (n)	Difference (mm)	p
ICpc	4.80 (18)	5.91 (18)	1.11	0.16 ns
ICc	4.35 (11)	5.91 (16)	1.56	0.06 ns
IPMdpc	6.46 (19)	7.11 (16)	0.65	0.48 ns
IPMdc	5.90 (19)	6.65 (16)	0.75	0.29 ns
IMppc	6.11 (24)	7.55 (22)	1.44	0.02*
IMpc	5.24 (24)	6.31 (21)	1.07	0.08 ns

ns = non-significant.

* statistically significant difference (p < 0.05).

## DISCUSSION

Cleft lip and palate occurs in the mid face and causes structural problems in the alveolar bone and maxilla. The urgent relationship with the malocclusion is demonstrated by the anatomic rupture that compromises the alveolar ridge as well as dental problems, such as agenesis and malpositioning, as well as sagittal and transverse maxillary difficiencies.^10^ The treatment protocol adopted by the Hospital for Rehabilitation of Craniofacial Anomalies/USP for patients with unilateral complete cleft lip and palate emphasizes early surgical procedures (cheiloplasty and palatoplasty), with no orthopedic intervention in the preoperative or immediate postoperative periods. As a general rule, orthodontic treatment is initiated at the onset of the mixed dentition phase. Two reasons justify the lack of orthodontic intervention in the deciduous dentition phase: the instability of the early correction of cross bites, which leads to an excessively long treatment and retention time; and the fact that alterations in the shape of the dental arch and the occlusion are exacerbated in the mixed dentition phase.^11^

Expansion is the first step of orthodontic treatment (pre-alveolar bone graft). It aims at reestablishing the transverse dimensions of the atresic maxilla. The maxillary expander designed by Haas is the main appliance used for lateral repositioning of the collapsed maxillary processes^6^ and follows the same activation protocol used for patients without cleft palate. In this phase, the inverse traction of the maxilla may be associated with the post-expansion period to revert cases of negative sagittal discrepancy, expressed by anterior crossbite.^12^ The Hyrax appliance is also commonly used for maxillary expansion. The main difference between the two expanders is the acrylic support on the palate in the Haas appliance. However, both expanders are effective in increasing the transverse dimension of the maxilla. The choice of one over the other is based on the shape of the patient's palate. Whenever the transverse width and depth allow, the Haas expander is the appliance of choice due to the anchorage provided by its acrylic portion. In the present study, the modified Haas expander was used, which differs from the original by the presence of two orthodontic bands instead of four, and by bonded orthodontic clips on the deciduous molars.

Following the tendency of using digital records in Orthodontics,^13^ the evaluation of the transverse effect of the Haas and Hyrax expanders was performed with digital models, which offer advantages in terms of storage, retrieval, durability, diagnostic versatility and transmitting information.^14^ Moreover, studies comparing digital and conventional models report considerable accuracy and reproducibility in the measurements of tooth width, overjet and overbite.^15^ The ICC of the measurements obtained from the digital models by a single examiner on two different occasions demonstrate the reliability of the method (Table 1).

Both appliances were capable of restoring the adequate upper arch morphology and correcting the posterior cross bite (Figs 1 and 2). The results demonstrate significant increase (p < 0.0001) in all transverse dimensions measured (Tables 2 and 3), which is in agreement with findings reported in the literature.^16^

The increases in the inter-molar, inter-premolar or inter-deciduous molar and inter-canine widths were statistically significant in both groups. The group treated with the Haas expander had an increase in inter-molar distance of 6.11 mm on the tips of the mesiovestibular cusps, 5.24 mm on the palatine-cervical portion of the molars, and an increase in the inter-canine distance of 4.79 mm when measured on the cusps and 4.35 mm when measured on the palatine-cervical portion (Table 2). These values are similar to those reported by previous studies.^17,19^ In a study involving digital models of 32 children without cleft palate and with unilateral or bilateral cross bite (16 children treated with the Haas expander and 16 treated with the Hyrax expander),the Haas group had an increase in inter-molar distance of 6.33 mm on the tips of the mesiovestibular cusps and 6.04 mm on the central sulcus of the first molars; an increase in the inter-canine distance of 2.27 mm when measured on the cusps and 4.74 mm when measured on the cervical portion.^20^

In the present study, the patients treated with the Hyrax expander had an increase in inter-molar distance of 7.55 mm on the tips of the mesiovestibular cusps and 6.31 mm in the palatine-cervical portion; an increase in the inter-canine distance of 5.91 mm when measured on the cusps or on the palatine-cervical portion (Table 3). These results are similar to findings reported in the literature for patients in the mixed dentition phase treated with the Hyrax expander.^19,21,23^ However, another study reports larger increases (9.97 mm in inter-molar distance on the tips of the mesiopalatine cusps and 9.51 mm in the in the mesial portion of the central sulcus of the and 7.93 mm in the inter-canine distance when measured on the cusps and 6.29 mm when measured on the cervical portion).^20^

No statistically significant differences were found between groups in the comparison of the inter-molar and inter-canine distances obtained with the Haas or Hyrax appliances, except for the inter-molar distance measured on the tips of the cusps, which was greater in the Hyrax group (Table 4). This was likely due to the greater tooth tipping caused by this appliance. Discrepant results are reported in the literature, with some studies reporting a greater increase in inter-molar and inter-canine distances using the Hyrax expander^20^ and others reporting a greater tendency of vestibular tipping of molars using the Haas expander.^16^ Both appliances generally demonstrate similar behavior regarding the expansion of the dentoalveolar region of the maxilla.^24^ However, the Haas expander may cause greater vestibular tipping of the anchoring teeth (3.5º for the first molar) in comparison to the Hyrax expander (1.6º), although this is not a clinically relevant difference.^24^

## CONCLUSIONS

- Rapid maxillary expansion using the modified Haas and Hyrax expanders proved efficient in increasing the transverse dimensions of the upper dental arch in patients with unilateral complete cleft lip and palate.
- No significant differences between appliances were found regarding the transverse effects produced by the modified Haas and Hyrax expanders in the present study.
